# Preconditioning human natural killer cells with chorionic villous mesenchymal stem cells stimulates their expression of inflammatory and anti-tumor molecules

**DOI:** 10.1186/s13287-019-1153-9

**Published:** 2019-02-06

**Authors:** M. H. Abumaree, N. A. Alshehri, A. Almotery, A. M. Al Subayyil, E. Bahattab, F. M. Abomaray, T. Khatlani, B. Kalionis, D. Jawdat, M. F. El-Muzaini, M. A. Al Jumah, A. S. AlAskar

**Affiliations:** 1 Stem Cells and Regenerative Medicine Department, King Abdullah International Medical Research Center, King Abdulaziz Medical City, Ministry of National Guard Health Affairs, P.O. Box 22490, 11426, Mail Code 1515, Riyadh, Saudi Arabia; 2 College of Science and Health Professions, King Saud Bin Abdulaziz University for Health Sciences, King Abdulaziz Medical City, Ministry of National Guard Health Affairs, P.O. Box 3660, 11481, Mail Code 3124, Riyadh, Saudi Arabia; 3 College of Applied Medical Sciences, King Saud Bin Abdulaziz University for Health Sciences, P.O. Box 3660, 11481, Mail Code, Riyadh, 3124 Saudi Arabia; 4 National Center for Stem Cell Technology, Life Sciences and Environment Research Institute, King Abdulaziz City for Science and Technology, P.O Box 6086, Riyadh, 11442 Saudi Arabia; 5 Department of Clinical Science, Intervention and Technology, Division of Obstetrics and Gynecology, Karolinska Institutet, 14186 Stockholm, Sweden; 6 Center for Hematology and Regenerative Medicine, Karolinska Institutet, 14186 Stockholm, Sweden; 7 Department of Maternal-Fetal Medicine Pregnancy Research Centre, Department of Obstetrics and Gynaecology, Royal Women’s Hospital, University of Melbourne, Parkville, Victoria 3052 Australia; 8 Department of Obstetrics and Gynaecology, King Abdulaziz Medical City, Minstry of National Guard Health Affairs, P.O. Box 3660, 11481, Mail Code, Riyadh, 3124 Saudi Arabia; 9 College of Medicine, King Saud Bin Abdulaziz University for Health Sciences, King Abdulaziz Medical City, Ministry of National Guard Health Affairs, P.O. Box 3660, 11481, Mail Code, Riyadh, 3124 Saudi Arabia; 10 Adult Hematology and Stem Cell Transplantation, King Abdulaziz Medical City, Ministry of National Guard Health Affairs, P.O. Box 22490, 11426, Mail Code, Riyadh, 1515 Saudi Arabia

**Keywords:** Chorionic villous mesenchymal stem/multipotent stromal cells, NK cells, Cytolytic activity, NK cell proliferation, Cancer, Inflammatory molecules

## Abstract

**Background:**

Mesenchymal stem cells derived from the chorionic villi of human placentae (pMSCs) produce a unique array of mediators that regulate the essential cellular functions of their target cells. These properties make pMSCs attractive candidates for cell-based therapy. Here, we examined the effects of culturing human natural killer (NK) cells with pMSCs on NK cell functions.

**Methods:**

pMSCs were cultured with IL-2-activated and non-activated NK cells. NK cell proliferation and cytolytic activities were monitored. NK cell expression of receptors mediating their cytolytic activity against pMSCs, and the mechanisms underlying this effect on pMSCs, were also investigated.

**Results:**

Our findings show that IL-2-activated NK cells, but not freshly isolated NK cells, efficiently lyse pMSCs and that this response might involve the activating NK cell receptor CD69. Interestingly, although pMSCs expressed HLA class I molecules, they were nevertheless lysed by NK cells, suggesting that HLA class I antigens do not play a significant role in protecting pMSCs from NK cell cytolytic activity. Co-culturing NK cells with pMSCs also inhibited NK cell expression of receptors, including CD69, NKpG2D, CD94, and NKp30, although these co-cultured NK cells were not inhibited in lysing cancer cells in vitro. Importantly, co-cultured NK cells significantly increased their production of molecules with anti-tumor effects.

**Conclusions:**

These findings suggest that pMSCs might have potential applications in cancer therapy.

## Background

Natural killer cells (NK cells) are lymphocytes, which function in the innate immune system, and have cytotoxic or cytolytic activities that are specifically targeted against virally infected cells and tumor cells [1]. The cytolytic activities of NK cells on target cells are mediated by several cell surface-activating receptors (including NKp30, NKp44, NKp46, DNAM-1, and NKG2D) and inhibitory receptors (including the killer immunoglobulin-like receptors “KIRs” and CD94/NKG2A) [1–11], while other NK cell functions are mediated by a range of mediators including cytokines and their corresponding receptors, as well as their expression of Toll-like receptors (TLRs) [1, 12, 13].

The activating receptors mediate the cytolytic activity of NK cells by binding to their corresponding ligands [poliovirus receptor (PVR) and Nectin-2 for DNAM-1, as well as MICA/B (MHC class I chain-related genes A and B) and ULBPs (UL16-binding proteins) for NKG2D] on target cells [2, 3, 6–11]. NK inhibitory receptors, specifically KIRs and CD94/NKG2A, inhibit the cytolytic activity of NK cells against their target cells following their interaction with human leukocyte antigen (HLA) class I and non-classical MHC class I (HLA-E) molecules, respectively [4, 5]. In contrast, when NK cells interact with target cells that lack HLA molecules, NK cells are activated and this subsequently induces the lysis of target cells [14].

The interaction between human NK cells and mesenchymal stem/stromal cells (MSC) has been recently reported by us and others [15–19]. MSCs are multipotent, adult stem/stromal cells and with the ability of self-renewal and differentiation into the three mesenchymal lineages of adipocyte, osteocyte, and chondrocyte [20–23]. MSCs can be isolated from various adult tissues, such as human placenta [20–23]. We recently reported that the interaction between NK cells and MSCs from the maternal side of human placenta known as *decidua parietalis* (DPMSCs) results in the lysis of DPMSCs [19]. Similarly, NK cells can also lyse human bone marrow-derived MSCs (BMMSCs) [15–18].

Previously, we isolated MSCs from the fetal part of human term placenta known as chorionic villi [23]. These placental MSCs (pMSCs) have immunosuppressive properties [23–25]. pMSCs induce the differentiation of anti-inflammatory macrophages (M2 macrophages) from human monocytes [25] and exert inhibitory effects on the functions of human dendritic and T cells [26]. Thus, pMSCs can control the functions of immune cells that mediate both the innate and adaptive immune responses. These properties make pMSCs attractive candidates for cell-based therapy.

The principle for the successful use of pMSCs as a cell-based therapy is to have a full description of their interaction with a wide range of immune cells. Currently, the consequences of the interaction between pMSCs and human NK cells are unknown. Therefore, we conducted this study to investigate the interactions between pMSCs and NK cells and the outcomes of this interaction. We found that pMSCs inhibit the proliferation of both resting non-activated NK cells (NK cells induced to proliferate by IL-2) and activated NK cells (NK cells pre-activated by IL-2). We also found that IL-2-activated NK cells produce a strong cytolytic response against pMSCs and that this response might involve the activating NK cell receptor CD69. pMSCs did not alter NK cell cytolytic activity against cancer cells; however, most important was that pMSCs induced NK cell expression of several molecules with anti-tumor properties.

## Methods

### Ethics and collection of human placentae and peripheral blood

This study was approved by the institutional research board (IRB), King Abdulla International Medical Research Centre (KAIMRC), Saudi Arabia. Placentae from uncomplicated human term pregnancies (38–40 weeks of gestation) and peripheral blood samples from healthy adult subjects were collected and processed immediately after consenting donors.

### Isolation and culture of pMSCs

MSCs from chorionic villi of human term placenta (pMSCs) were isolated using our published method [23]. Briefly, small pieces (~ 40 mg total wet weight) from the fetal chorionic villi underneath the layer of maternal decidua of the placental tissue were washed thoroughly with sterile phosphate buffered saline (PBS, pH 7.4) and then incubated in a digestion solution of DMEM-F12 (Dulbecco’s modified Eagle medium nutrient mixture F-12) medium (Life Technologies, Grand Island, USA) containing 2.5% trypsin (Life Technologies), 270 unit/mL DNase (Life Technologies), and antibiotics (100 U/L penicillin and 100 μg/mL streptomycin). After gentle rotation overnight at 4 °C, tissues were washed thoroughly with PBS, and the explant tissues were then cultured in a complete DMEMF-12 culture medium containing 10% mesenchymal stem cell certified fetal bovine serum (MSC-FBS) (Life Technologies), 100 μg/mL of l-glutamate, and the antibiotics described above. Tissues were then incubated at 37 °C in a humidified atmosphere containing 5% CO_2_ (a cell culture incubator). When cells migrated out of the explants, they were harvested with TrypLE™ Express detachment solution (Life Technologies) and then characterized by flow cytometry using MSC markers and hematopoietic markers (Table 1) and they were also evaluated for differentiation into adipocytes, chondrocytes, and osteocytes using adipogenic as previously published [23]. pMSCs (passage 2) from twenty placentae were used in this study.

**Table 1:** Monoclonal antibodies used in this study to characterize pMSCs and NK cells

Markers	Monoclonal antibodies								
MSC positive markers	CD44	CD90	CD105	CD146	CD166	HLA-ABC			
Hematopoietic markers	CD14	CD19	CD40	CD45	CD80	CD83	CD86	HLA-DR	
NK cell marker	CD56								
NK cell-activating receptors	CD69	CD226	NKp30	NKp44	NKp46	NKpG2D (CD314)			
NK cell inhibitory receptor	CD94/NKG2A								
NK cell receptor ligands	PVR (CD155)	Nectin-2 (CD112)	ULBP-1	ULBP-2	ULBP-3	MICA	MICB	HLA-E	
Immune proteins	IL-12Rβ1	IL18Rα	IL-18Rβ	IFN-ɣR1	IFN-ɣR2	TLR3	TLR7	TLR9	TNF-α

### Isolation of human NK cells

NK cells were isolated from mononuclear cells of a peripheral blood (PBMNCs) obtained from ten healthy donors as previously described by us [19]. Trypan blue was used to determine the viability of NK cells, while NK cell purity was assessed using anti-CD56 monoclonal antibody (R & D Systems, Abingdon, UK) in a flow cytometry. The viability and purity of NK cells used in this study were more than 95%. After isolation, NK cells were used either immediately or after 72 h activation with 100 U/mL IL-2 (R & D Systems) in RPMI 1640 medium (Life Technologies) containing 10% FBS, 2 mM l-glutamine and the antibiotics described above (NK culture medium).

### NK cell proliferation assay using a tetrazolium compound [3-(4, 5-dimethylthiazol-2-yl)-5-(3-carboxymethoxyphenyl)-2-(4-sulfophenyl)-2H-tetrazolium, inner salt] MTS

An MTS kit (catalogue number G5421, Promega, WI, USA) was used to evaluate NK cell proliferation as previously described by us [19]. Briefly, resting non-activated NK cells (NK cells induced to proliferate by culturing with 100 U/mL IL-2) or activated NK cells (NK cells pre-cultured with 100 U/mL IL-2 for 72 h) as we previously published [19] were cultured with or without pMSCs at different ratios of NK to pMSC. The ratios of NK to pMSC ranged from 1:1, 1:5, 1:10, 1:25, 1:50, and 1:100. NK culture medium (described above) was used in all NK cell-pMSC co-culture experiments. Prior to the addition of pMSCs to NK cells in proliferation experiments, pMSCs were pre-incubated with mitomycin C to inhibit pMSC proliferation as previously described [27]. After 3 days of culture, NK cell proliferation was determined by incubating cells in MTS solution as we previously published [9]. Results from triplicate samples were presented as means (± standard errors). Experiments were performed in triplicate and repeated ten times using ten individual preparations of both pMSCs and NK cells.

### NK cell cytolytic experiments

To determine the cytolytic potential of NK cells against pMSCs, IL-2-non-activated NK cells, and activated NK cells (NK cells pre-cultured with 100 U/mL IL-2 for 72 h), were cultured with pMSCs at different ratios of NK to pMSC (i.e., NK to pMSC ratios of 1:1, 15:1, 25:1, 50:1, and 100:1). NK cell activation was carried out as previously described by us [19]. Briefly, adherent pMSCs were co-cultured with NK cells (IL-2-activated and non-activated NK cells) at different ratios of NK to pMSC (as indicated above) and then incubated at 37 °C. For blocking assays, NK cells were preincubated with monoclonal antibody specific to different NK cell activating and inhibitory receptors (Table 2) at 10 μg/mL (final concentration) as previously described by us [19]. After incubation for 30 min at 4 °C, NK cells were washed and then used in the cytolytic experiments at 25:1 NK to pMSC ratio. pMSC surface expression of ligands recognized by NK cell activating or inhibiting receptors (Table 1) was evaluated using flow cytometry as previously described by us [19]. Controls were NK cells and pMSCs, where each cell type is cultured alone. Cell lysis was evaluated as previously described by us [19]. The viability of pMSCs was determined by the Trypan blue exclusion assay. Experiments were carried out and repeated using preparations of pMSCs and NK cells as described above.

**Table 2:** Monoclonal antibodies used in the blocking experiments

Monoclonal antibody	NKp30	NKp44	NKp46	CD69	CD226 (DNAM-1)	NKG2D (CD314)	CD94
Isotype	IgG_2A_	IgG2a	IgG2b	IgG2a	IgG1	IgG1	IgG1
Clone	210847	253415	195314	298633	102511	149810	131412
Catalog number	MAB 1849	MAB22491	MAB 1850	MAB23591	MAB666	MAB139	MAB1058

### NK cell cytolytic activity against tumor cells

To study the NK cell cytolytic activity following their incubation with pMSCs for 24 h against MCF-7 cells (breast cancer cells, ATCC, Manassas, VA, USA), our previously published method was used [19]. Briefly, NK cells [IL-2-activated NK cells cultured alone, or co-cultured with pMSCs, at NK to pMSC ratios of 15:1, 25:1, 50:1, and 100:1 in the NK/pMSC cytolytic experiment described above] were harvested and then purified as described above. CD56^+^ NK cells with viability and purity of more than 95% were used against MCF-7 cells at an NK to MCF-7 ratio of 10:1. 10NK to 1MCF-7 ratio was used, because it completely lyzed MCF-7 cells as previously reported by us [19]. Cells were cultured in NK culture medium and then incubated at 37 °C. Cell lysis was then evaluated as described above. Controls were IL-2-treated NK cells cultured without pMSCs served as a control or MCF-7 cells cultured alone. The viability and purity of NK cells were determined as described above. Triplicate experiments were carried out and repeated ten times using NK cells isolated from ten independent pMSC/NK cytolytic assays and MC7 breast cancer cells.

### NK cell cytolytic activity against pMSCs and tumor cells using xCELLigence real-time system

The cytolytic activities of NK cells against pMSCs or MCF-7 were also determined using a real-time cell analyzer system (xCELLigence RTCA-DP, Roche Diagnostics, Mannheim, Germany) as we and other previously described [19, 28]. For the NK/pMSC cytolytic experiment, NK cells [IL-2-non-activated or activated NK cells (as described above)] were used against pMSCs at different ratios (NK to pMSC ratios of 1:1, 15:1, 25:1, 50:1, and 100:1). For the NK/MCF-7 cytolytic experiment, NK cells [IL-2-activated NK cells cultured alone or with pMSCs in the NK/pMSC cytolytic experiment (see above)] were used against MCF-7 cells. After incubation of NK cells with pMSCs (see above), NK cells were isolated and their viability as well as purity was assessed as described above, and then used against MCF-7 cells at a NK to MCF-7 ratio of 10:1. For each experiment, 20 × 10^3^ pMSCs or MCF-7 cells, with or without NK cells at the indicated ratios, were seeded in NK culture medium in quadruplicate wells of 16-well E-culture plates (catalog number 05469813001, Roche Diagnostics), and the cell growth of the pMSC/NK or MCF-7/NK cultures was monitored in a real time as previously described by us [19]. The rate of cell growth (cell index) was determined by calculating the linear regression of the slopes between two given time points using the xCELLigence software (version 1.2.1), and data was expressed as mean ± SD. Five experiments were carried out using pMSCs (P2) and NK cells from five independent preparations each.

### Quantitation of cytokines in culture supernatants

To examine the NK cell secretion of cytokines against pMSCs in the cytolytic experiments described above, the culture medium from the cytolytic experiment was collected after 24 h by centrifugation at 800×*g* for 10 min and then screened for several cytokines including interferon gamma (IFNγ), IL12, granulocyte-macrophage colony-stimulating factor (GM-CSF), IL1β, IL10, interleukin-1 receptor antagonist (IL-1Ra), and macrophage migration inhibitory factor (MIF)] using quantitative sandwich immunoassay. ELISA kits were purchased from R & D Systems, Life Technology and MyBioSource (California, USA). Complete RPM-1640 medium was included as a negative control. Experiments were carried out in duplicate and repeated ten times using ten individual preparations of both pMSCs and NK cells.

### NK cell expression of activating and inhibitory receptors and immune proteins

NK cell expression of activating and inhibitory receptors as well as immune proteins (Table 1) following their co-culture with pMSCs (see above) was determined by flow cytometry after harvesting them from the cytolytic assay and then purifying them as described above.

### Flow cytometry

Cells (1 × 10^5^) were incubated with antibodies listed in Table 1 for 30 min, and then flow cytometry for cell surface and intracellular proteins was performed as we previously described [19]. Negative controls were cells incubated with FITC or PE-labeled mouse IgG isotype antibody.

### Statistical analysis

GraphPad Prism 5 software (non-parametric tests (Mann-Whitney *U* and Kruskal-Wallis)) was used to analyze data, which were considered statistically significant if *P* < 0.05.

## Results

### pMSC isolation from human placental chorionic villi

pMSCs were isolated from human placental chorionic villi and cultured as previously described [23]. On the second passage of these cells, > 95% were positive for MSC markers and negative (i.e., < 5% positive) for hematopoietic markers (data not shown), supporting previously published results [23]. In addition, pMSCs differentiated into adipocytes, osteocytes, and chondrocytes, as previously reported (data not shown) [23]. On the basis of these findings, second-passage pMSCs were used in all pMSC experiments reported in this study.

### pMSCs inhibit NK cell proliferation

**Fig. 1:**
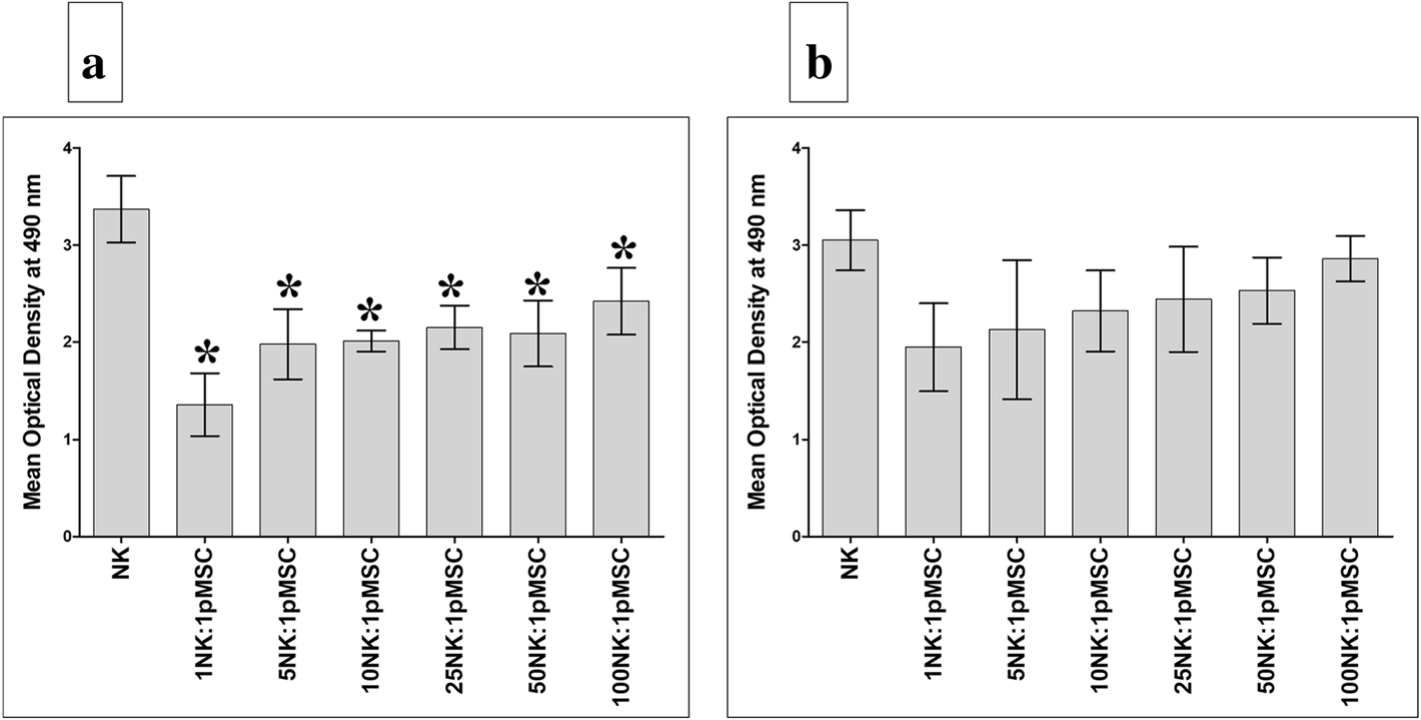
pMSC effect on NK cell proliferation. **a** Proliferation of resting unactivated cells (NK cells were induced to proliferate by 100 U/mL IL-2) was significantly decreased in the presence of pMSCs as compared to untreated resting unactivated NK cells, *P < 0.05. **b** IL-2-activated NK cell (NK cells were initially activated by 100 U/mL IL-2 for 72 h) proliferation decreased, but not statistically significant, in the presence of pMSCs as compared to untreated IL-2-activated NK cells, *P* > 0.05. Results of ten representative experiments in which the proliferation of resting unactivated or IL-2-activated NK cells cultured for 72 h with or without Mitomycin C-treated pMSCs at different ratios of NK to pMSC ranging from 1:1, 5:1, 25:1, 50:1, and 100:1 was measured using the MTS proliferation method. Experiments were conducted in triplicate using NK cells and pMSCs prepared from the peripheral blood of ten different healthy subjects and ten different normal human term placentae, respectively. Bars represent standard errors

In a previous study, we found that human pMSCs significantly inhibited T cell proliferation [26]. To investigate whether pMSCs also inhibit NK cell proliferation, pMSCs were cultured with resting non-activated or activated NK cells. After 72 h in culture, pMSCs significantly inhibited the proliferation of resting NK cells at all NK to pMSC ratios investigated (NK to pMSC ratios of 1:1 to 100:1; *P* < 0.05, Fig. 1a). In contrast, pMSCs were less efficient in inhibiting NK cells following their stimulation, *P* > 0.05 (Fig. 1b).

### pMSCs express ligands that bind NK cell receptors

To investigate whether NK cells and pMSCs interact, we examined whether pMSCs express the ligands that bind to various activating and inhibiting NK cell receptors, using flow cytometry. The percentage of pMSCs that expressed ligands for the NK activating receptors, DNAM-1 and NKG2D, and for the NK inhibitory receptors, CD94/NGK2, is shown in Table 3. The most highly expressed NK activating receptor ligand by pMSCs was ULBP-2, which binds to the NKG2D receptor (37.33% ± 10.27%), and the least expressed ligand was PVR, which binds to DNAM-1 (12.17% ± 1.92%). In addition, 30.93% (± 8.36%) of pMSCs expressed HLA-E, and 95.60% (± 3.40%) expressed HLA-ABC, which are both ligands for the NK cell inhibitory receptors.

**Table 3:** Percentage of cells expressing surface ligands of NK activating receptors (DNAM-1 and NKG2D), and the ligands of NK cell inhibiting receptor (HLA-E) in different pMSCs

Receptors/molecule	Ligand names	Results (%)
DNAM-1	PVR (CD155)	12.17% ± 1.92%
	Nectin-2 (CD112)	1.73% ± 0.63%
NKG2D	ULBP-1	7.66% ± 3.28%
	ULBP-2	37.33% ± 10.27%
	ULBP-3	9.00% ± .5.56%
	MICA	6.33% ± 2.33%
	MICB	3.330% ± 1.45%
CD94/NKG2	HLA-E	27.33% ± 9.26%
HLA-ABC		95.00% ± 4.40%

### pMSC lysis by NK cells

**Fig. 2:**
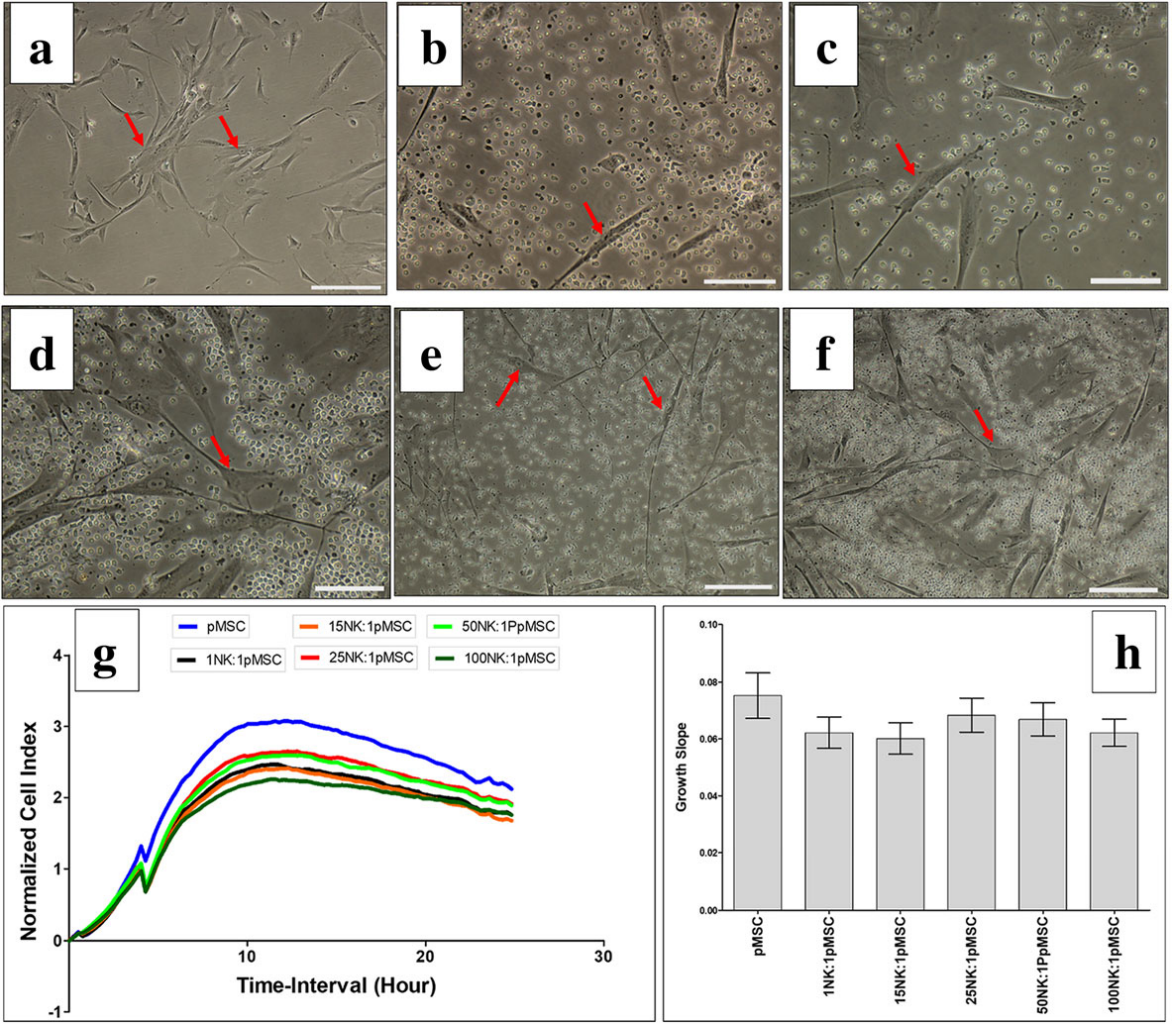
NK cell interaction with pMSCs evaluated by culturing IL-2 unactivated NK cells with pMSCs at different NK to pMSC ratios for 24 h and NK cell cytolytic activity against pMSCs were then evaluated using microscopic examination and the xCELLigence real-time cell system. Representative phase contrast microscopic images showing untreated pMSCs (control) have a spindle-like morphology (arrow) (**a**), unlysed pMSCs cultured with IL-2-unactivated NK cells (arrow) at ratio 1NK to 1pMSC (**b**), 15NK to 1pMSC (**c**), 25NK to 1pMSC (**d**), 50NK to 1pMSC (**e**), and 100NK to 1pMSC (**f**). The results of the xCELLigence showing that after 24 h culture, IL-2-unactivated NK cells did not lyse pMSCs as reflected by the cell index (**g**) and decreased pMSC proliferation (growth slope), but statistically was not significant, *P* > 0.05 (**h**) at all examined NK to pMSCs ratios. Experiments were carried out in triplicate and repeated for ten times using NK cells and pMSCs prepared from the peripheral blood of ten different healthy donors and ten different normal human term placentae, respectively. Scale bars, 50 μm

**Fig. 3:**
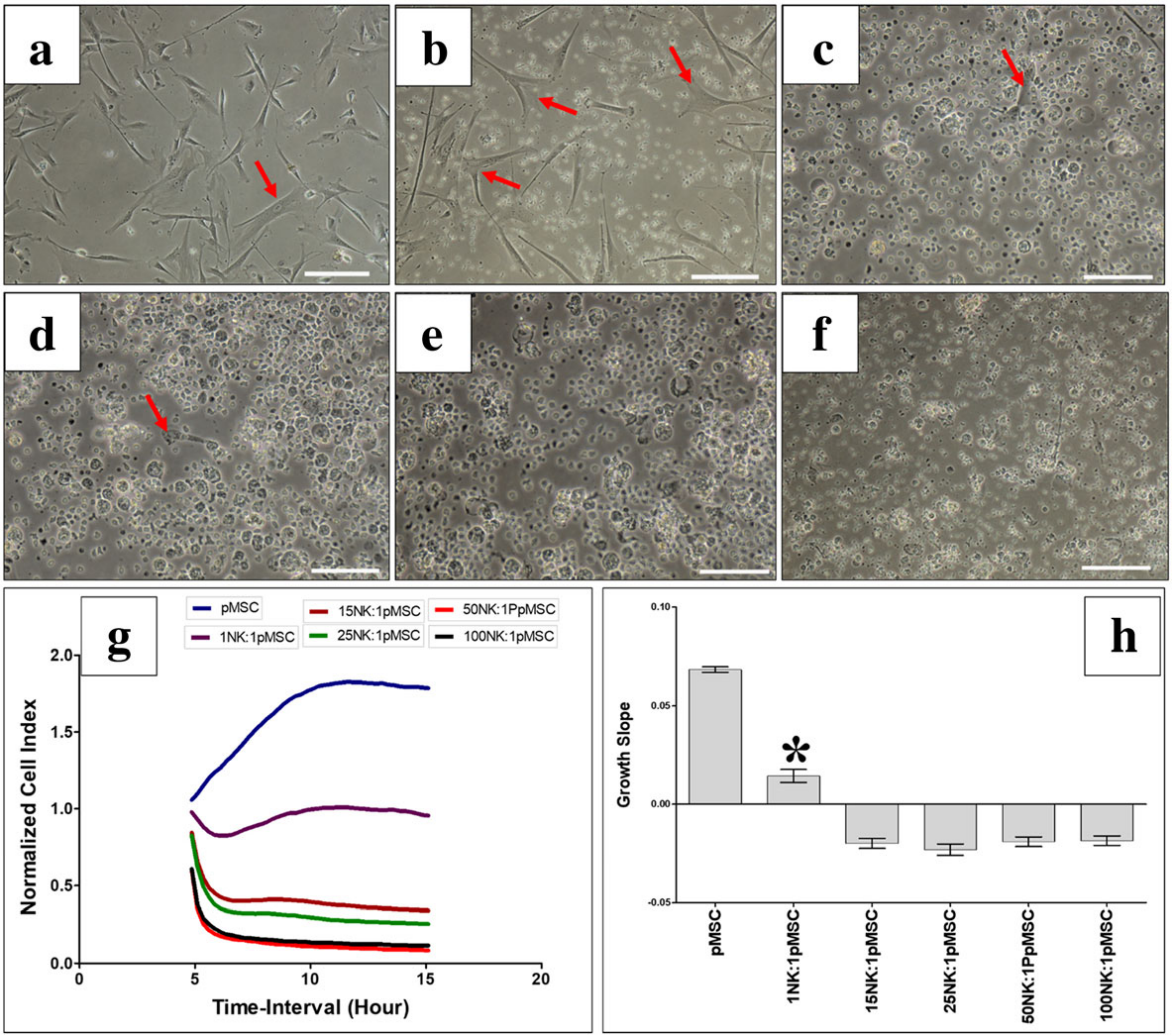
NK cell interaction with pMSCs evaluated by culturing IL-2-activated NK cells with pMSCs at different NK to pMSCs ratios and NK cytolytic activity against pMSCs were then evaluated within 24 h culture using microscopic examination and xCELLigence real-time cell system. Representative phase contrast microscopic images showing untreated pMSCs (control) with typical spindle-like morphology (arrow) (**a**), unlysed pMSCs cultured with IL-2-activated NK cells (arrow) at 1NK to 1pMSC ratio (**b**), lysed pMSCs cultured with IL-2-activated NK cells at ratios 15NK to 1pMSC (**c**), 25NK to 1pMSC (**d**), 50NK to 1pMSC (**e**), and 100NK to 1pMSC (**f**). The lysis of pMSCs was evident as no sign of intact adherent pMSCs, and cells were ruptured as cellular debris were evident in suspension. The results of the xCELLigence showing that after 15 h culture, IL-2-stimulated NK cells did not lyse pMSCs at 1NK to 1pMSC ratio, but pMSCs were lysed at 15NK to 1pMSC, 25NK to 1pMSC, 50NK to 1pMSC, and 100NK to 1pMSC as reflected by the cell index, which showing the cell index was almost zero indicating that pMSCs were not intact (no adhesion and proliferation) (**g**) and growth slope (**h**). At 1NK to 1pMSC ratio, pMSC proliferation significantly decreased, *P < 0.05 (**g**, **h**). Experiments were carried out in triplicate and repeated for ten times using NK cells and pMSCs prepared from the peripheral blood of ten different healthy donors and ten different normal human term placentae, respectively. Scale bars, 50 μm

The expression by pMSCs of ligands that activate NK cell receptors suggests that pMSCs could be susceptible to lysis by NK cells. Consequently, we examined the ability of NK cells, activated and not activated by IL-2, to lyse pMSCs. As previously reported by us and others [15, 18, 19], NK cells not activated by IL-2 were unable to lyse pMSCs (Fig. 2a–h). However, non-activated NK cells reduced pMSC proliferation at all NK to pMSC ratios examined (1:1, 15:1, 25:1, 50:1, and 100:1), but this reduction was not significant (*P* > 0.05; Fig. 2g, h). In contrast, following the activation of NK cells with IL-2 for 72 h, NK cells lysed pMSCs at all NK to pMSC ratios, except 1:1, within 24 h in culture. At these ratios, pMSCs were completely lysed by NK cells, as indicated by the lack of intact adherent pMSCs, as well as by the presence of ruptured cells and cellular debris in suspension within 24 h (Fig. 3c–f). The lysis of pMSCs by NK cells was also confirmed by the xCELLigence real-time cell system. The cell index, which represents the cell adhesion and proliferation of intact cells, was almost equal to zero in this assay, reflecting the lysis of cells (Fig. 3g, h). However, at the NK to pMSC ratio of 1:1, NK cells were unable to lyse pMSCs (Fig. 3b, g, h), but significantly reduced their growth (Fig. 3g, h).

### The NK cell receptors that contribute to pMSC lysis

**Fig. 4:**
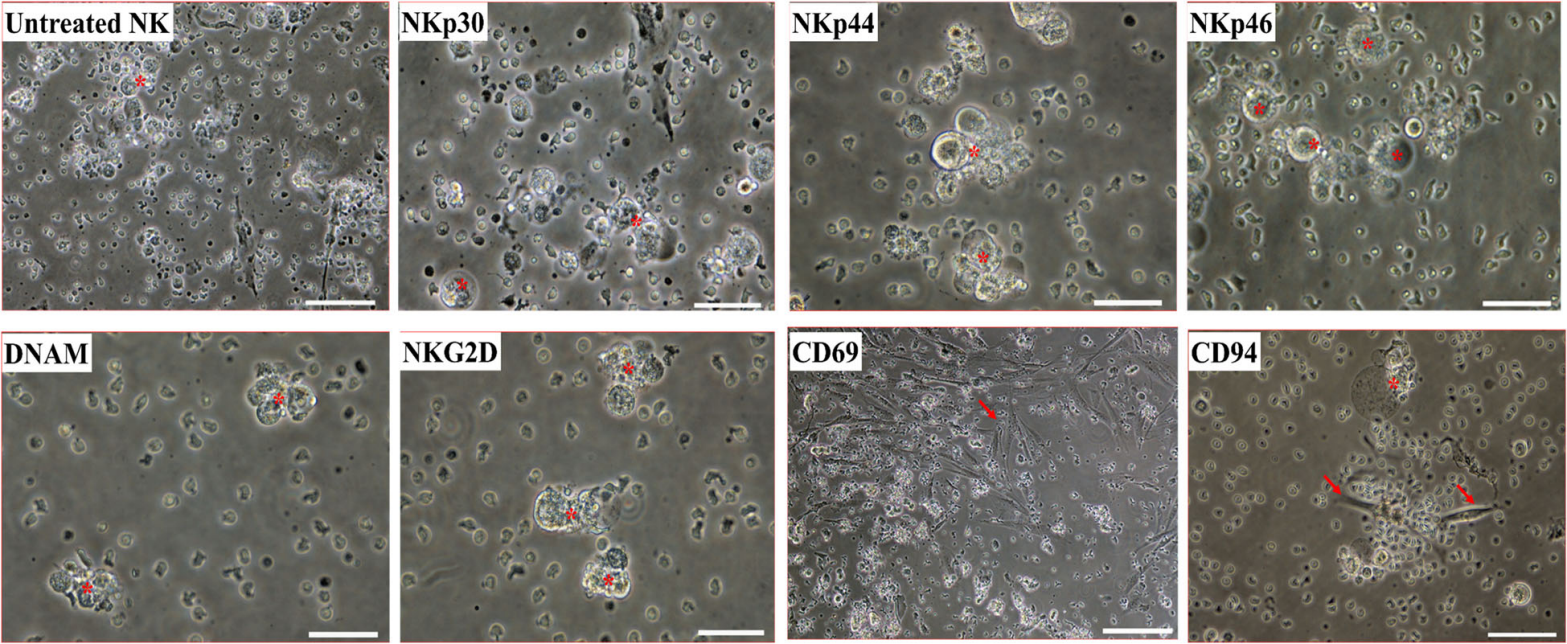
Antibody-mediated blocking experiments showing NK cell activating and inhibiting receptors mediating pMSC lysis. NK cells were pre-incubated with antibodies specific to indicated NK cell activating and inhibiting receptors; NK cells were then co-cultured with pMSCs at 25NK to 1pMSC ratio in a cytolytic experiment. pMSC lysis by NK cells was evaluated using microscopic examination within 24 h. Representative phase contrasts microscopic images showing complete lysis of pMSCs as no sign of intact adherent pMSCs and cells were also ruptured as cellular debris were evident in suspension (star pointing at adherent pMSCs) by untreated NK cells (NK cells were initially activated with 100 U/mL IL-2 for 72 h) by NKp30, by NKp44, NKp46, by DNAM, and by NKG2D and substantial inhibition of lysis of pMSC (arrow pointing at adherent pMSCs) by CD69. Blocking CD94 receptor did not increase the cytolytic activity of NK cells. Experiments were carried out in triplicate and repeated for ten times using NK cells and pMSCs prepared from the peripheral blood of ten different healthy donors and ten different normal human term placentae, respectively. Scale bars, 50 μm

To determine which NK cell receptors mediate pMSC lysis by NK cells, we used the monoclonal antibody-mediated blocking method in cytolytic experiments, as previously described [19]. NK cells were tested against allogenic pMSCs. The antibody-mediated blocking of the receptors NKp30, NKp44, NKp46, DNAM, or NKG2D did not inhibit the lysis of pMSCs by NK cells, as indicated by the lack of intact adherent pMSCs (viability of pMSCs < 1%) and the presence of ruptured cells and cellular debris in suspension (Fig. 4). In contrast, the antibody-mediated blocking of the activating receptor CD69 resulted in a substantial inhibition of NK cell-mediated lysis of pMSCs (~ 75% of pMSCs were adherent; viability of pMSCs ~ 80% ± 5.69%), Fig. 4. However, blocking the HLA-E-specific inhibitory receptor CD94/NKG2A using this approach did not affect the NK cell cytolytic activity against pMSCs, viability of pMSC < 1% (Fig. 4).

### Functional activities of NK cells incubated with pMSCs

**Fig. 5:**
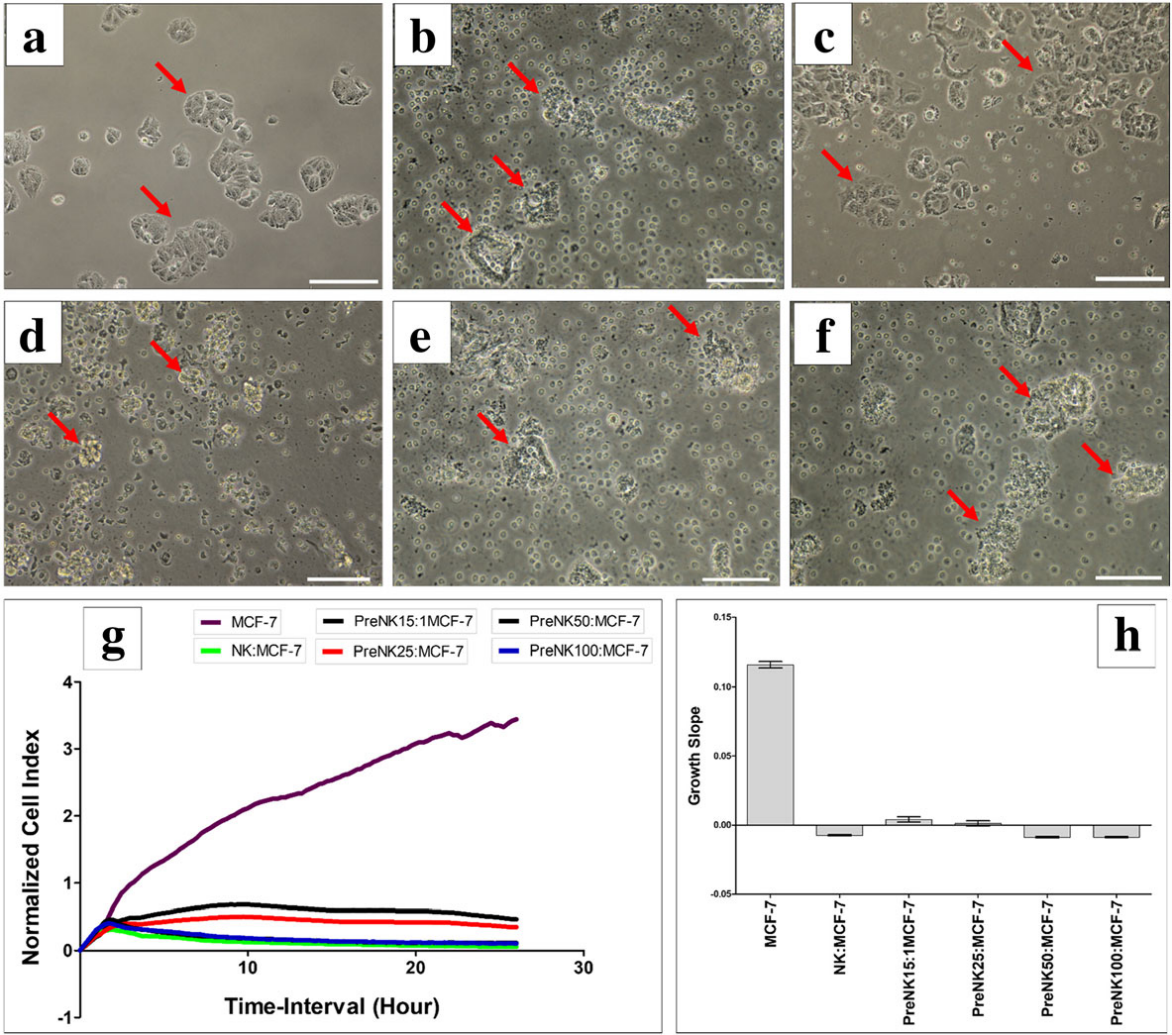
NK cell interaction with MCF-7 breast cancer cells evaluated by culturing IL-2-activated NK cells with pMSCs at different NK to pMSC ratios in a cytolytic experiment. Following 24 h incubation with pMSCs, NK cells were harvested, purified, and then added to MCF-7 cells at a 10NK to 1MCF-7 ratio, and NK cytolytic activity against MCF-7 cells was then evaluated using microscopic examination and the xCELLigence real-time cell system. Representative phase contrast microscopic images showing a complete lysis of MCF-7 cells (no sign of intact adherent MCF-7 and cells were also ruptured as cellular debris were evident in suspension, stars) by untreated NK cells (NK cells were initially activated with 100 U/mL IL-2 for 72 h) (**b**), pretreated NK cells (pretreated NK cells “pre-NK”: IL-2-activated NK cells precultured with pMSCs) at 15NK to 1pMSC (**c**), 25NK to 1pMSC (**d**), 50NK to 1pMSC (**e**), and 100NK to 1pMSC (**f**) as compared to MCF-7 cells (arrow) cultured alone (**a**) within 24 h culture. The results of the xCELLigence show that after 24 h culture, MCF-7 were completely lysed by untreated NK cells and treated NK cells (described above) as reflected by the cell index (**g**) and growth slope (**h**). The cell index was reduced almost to zero for MCF-7 co-cultured with NK cells indicating no sign of intact cells. Experiments were carried out in triplicate using the indicated NK to MC7 ratios using NK cells harvested from ten independent NK/pMSC cytolytic assays and MC7 breast cancer cells. Scale bars, 50 μm

The modulatory effects of pMSCs on NK cell cytolytic activity were further studied by co-culturing NK cells (NK cells were initially activated with IL-2 for 72 h) with pMSCs for 24 h at NK to pMSC ratios of 15:1, 25:1, 50:1, and 100:1. At these ratios and in these culture conditions, NK cells lysed pMSCs. The NK cells were then harvested, purified, and co-cultured with human MCF-7 breast cancer cells at a NK cell to MCF-7 ratio of 10:1, to test NK cell cytolytic activity. As shown in Fig. 5, compared to MCF-7 cells cultured alone (untreated), pMSC-untreated NK cells and pMSC-treated NK cells completely lysed MCF-7 cells, as indicated by the lack of intact adherent MCF-7 cells and by the presence of ruptured cells and cellular debris in suspension within 24 h. This cytolytic activity of NK cells against MCF-7 cells was also confirmed using the xCELLigence real-time system, which showed that the cell index for MCF-7 cells co-cultured with NK cells that had initially been cultured with pMSCs or cultured alone was zero, demonstrating MCF-7 cell lysis (Fig. 5g, h).

### The expression of activating and inhibiting receptors by NK cells exposed to pMSCs

**Fig. 6:**
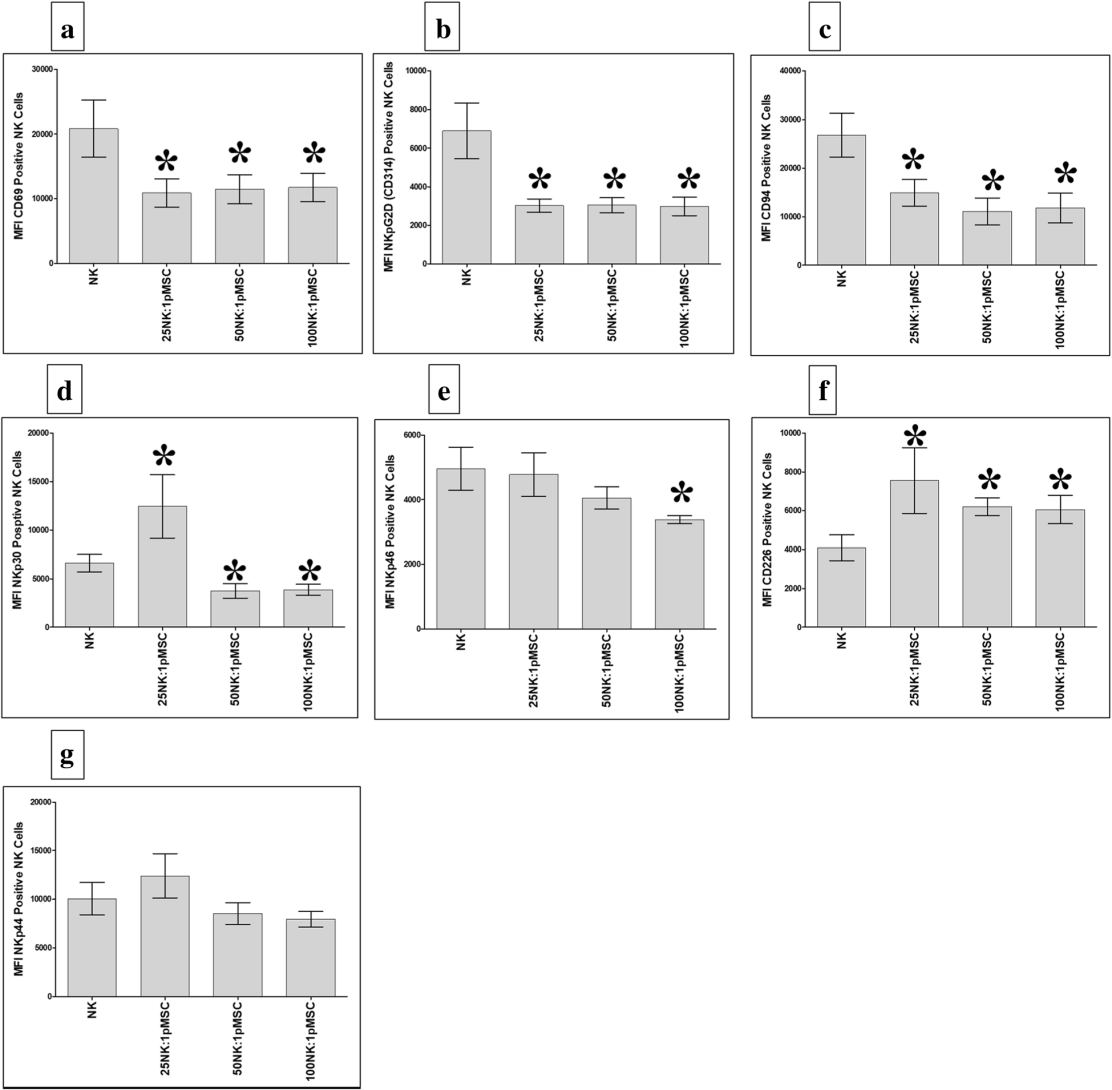
Flow cytometric analysis of indicated activating and inhibiting receptors by NK cells harvested from NK/pMSC cytolytic experiments. As compared with untreated NK cells, pMSCs significantly decreased NK cell expression of CD69 (**a**), NKG2D (**b**), and CD94 (**c**) at all NK to pMSC ratios (25:1, 50:1, and 100:1), *P < 0.05; significantly decreased NK cell expression of NKp30 at 25:1 and 50:1 NK to pMSC ratios, *P < 0.05 (**d**); significantly decreased NK cell expression of NKp46 at 100:1 NK to pMSC ratio, *P < 0.05 (**e**); significantly increased NK cell expression of DNAM (CD226) at all indicated NK to pMSC ratios, *P < 0.05 (**f**); and significantly increased NK cell expression of NKp30 at 25:1 NK to pMSC ratio, *P < 0.05 (**d**). pMSCs had no significant effect on NK cell expression of NKp44, *P* > 0.05 (**g**). Experiments were carried out in triplicate and repeated for ten times using ten independent NK/pMSC cytolytic experiments where in each experiment NK cells and pMSC were prepared from the peripheral blood of ten different healthy subjects and ten different normal human term placentae, respectively. Bars represent standard errors

To examine whether the phenotype of IL-2-activated NK cells was modified after being co-cultured with pMSCs in the above-described NK/pMSC cytolytic experiment, we evaluated the expression of several NK cell activating or inhibiting receptors using flow cytometry, and the expression was recorded as MFI. After 24 h in co-culture with pMSCs, IL-2-activated NK cells showed significantly decreased expression of the markers CD69, NKG2D, and CD94, relative to untreated NK cell controls, at all NK to pMSC ratios examined (25:1, 50:1, and 100:1; *P* < 0.05, Fig. 6a–c), while the expression of NKp30 was significantly decreased at the 50:1 and 100:1 NK to pMSC ratios (*P* < 0.05, Fig. 6d). In addition, the expression of NKp46 was significantly decreased in NK cells at the 100:1 NK to pMSC ratio (*P* < 0.05, Fig. 6e). In contrast, NK cell expression of DNAM (an activating receptor also known as CD226) was significantly increased at all NK to pMSC ratios (25:1, 50:1, and 100:1) relative to untreated NK cells (*P* < 0.05, Fig. 6f), while the expression of NKp30 significantly increased only at the 25:1 NK to pMSC ratio (*P* < 0.05, Fig. 6d). Co-culturing with pMSCs did not have a significant effect on the expression of NKp44 by NK cells (*P* > 0.05, Fig. 6g).

### The expression of immune markers by NK cells exposed to pMSCs

**Fig. 7:**
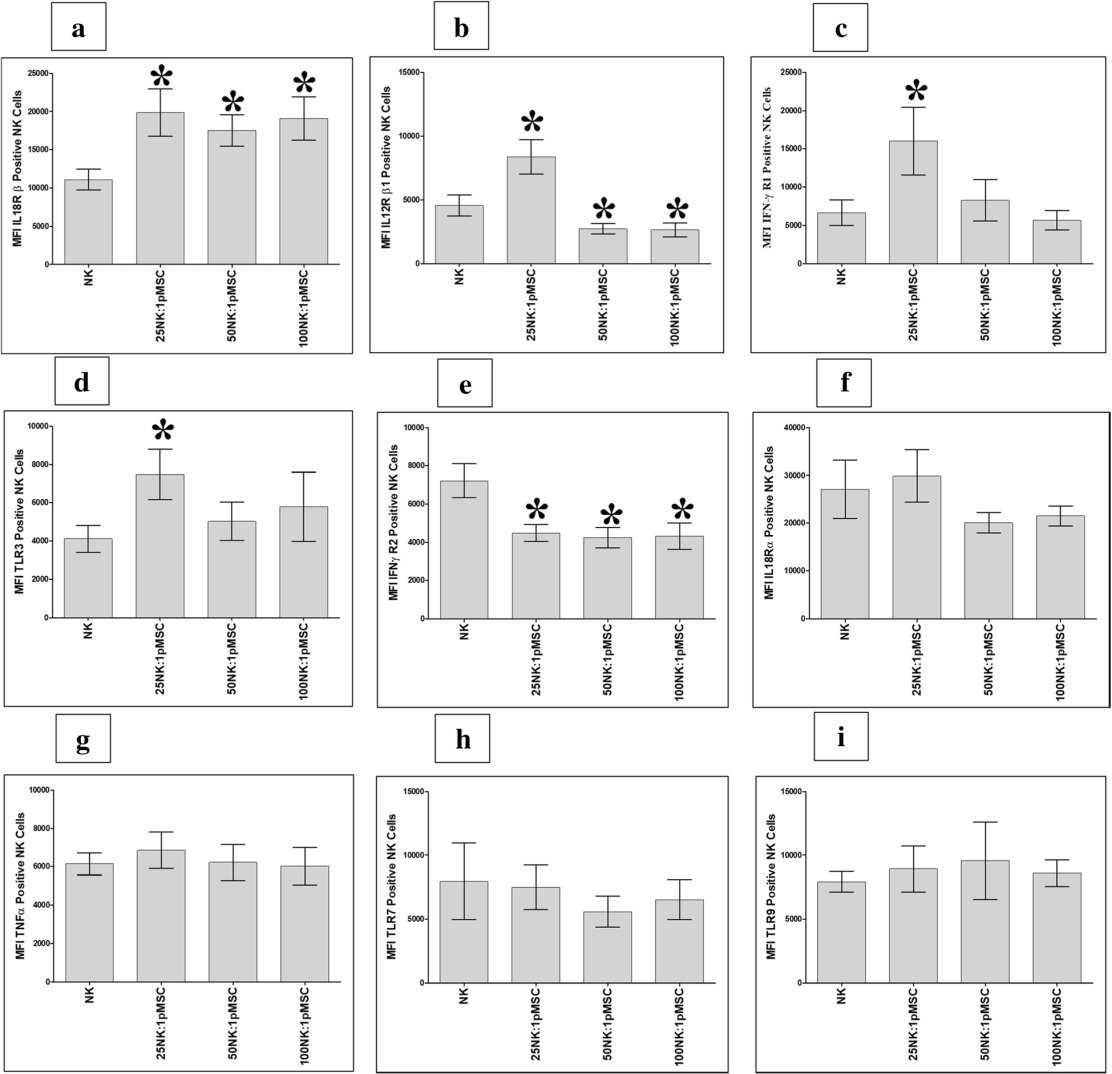
Flow cytometric analysis of indicated inflammatory molecules by NK cells harvested from NK/pMSC cytolytic experiments. As compared to untreated NK cells, pMSCs significantly increased NK cell expression of IL-18 Rβ at all indicated NK to pMSC ratios, *P < 0.05 (**a**); significantly increased NK cell expression of IL-12 Rβ1 (**b**), IFN-ɣ R1 (**c**), and TLR3 (**d**) at 25:1 NK to pMSC ratio, *P < 0.05; significantly decreased NK cell expression of IFN-ɣ R2 at all indicated NK to pMSC ratios, *P < 0.05 (**e**); and significantly decreased NK cell expression of IL-12 Rβ1 at 50:1 and 100:1 NK to pMSC ratios, *P < 0.05 (**a**). pMSC had no significant effect on NK cell expression of IFN-ɣ R1 (**c**) and TLR3 (**d**) at 50:1 and 100:1 NK to pMSC ratios, *P* > 0.05, and on IL-18 Rα (**f**), TNF-α (**g**), TLR7 (**h**), and TLR9 (**i**) at all indicated NK to pMSC ratios, *P* > 0.05. Experiments were carried out in triplicate and repeated for ten times using ten independent NK/pMSC cytolytic experiments where in each experiment NK cells, and pMSCs were prepared from the peripheral blood of ten different healthy subjects and ten different normal human term placentae, respectively. Bars represent standard errors

We also evaluated the NK cell expression of the immune markers (Table 1) after being co-cultured with pMSCs. After 24 h in culture with pMSCs, NK cell expression of IL-18Rβ significantly increased at all NK to pMSC ratios assessed (25:1, 50:1, and 100:1) compared to that of untreated NK cells (*P* < 0.05, Fig. 7a), while NK cell expression of IL-12Rβ1, IFN-ɣ R1, and TLR3 significantly increased at the 25:1 NK to pMSC ratio compared to that of untreated NK cells (*P* < 0.05, Fig. 7b–d). In contrast, compared to that of untreated NK cells, NK cell expression of IFN-ɣ R2 significantly decreased at all NK to pMSC ratios (*P* < 0.05, Fig. 7e), while NK cell expression of IL-12 Rβ1 significantly decreased at the NK to pMSC ratios of 50:1 and 100:1 (*P* < 0.05, Fig. 7b). In addition, co-culturing pMSCs with NK cells had no significant effect on the expression of IL-18 Rα, TNF-α, TLR7, and TLR9 at all NK to pMSC ratios, or on IFN-ɣ R1 and TLR3 at NK to pMSC ratios of 50:1 and 100:1, compared to that of untreated NK cells (*P* > 0.05, Fig. 7).

### pMSCs modulate NK cell secretion of cytokines

**Fig. 8:**
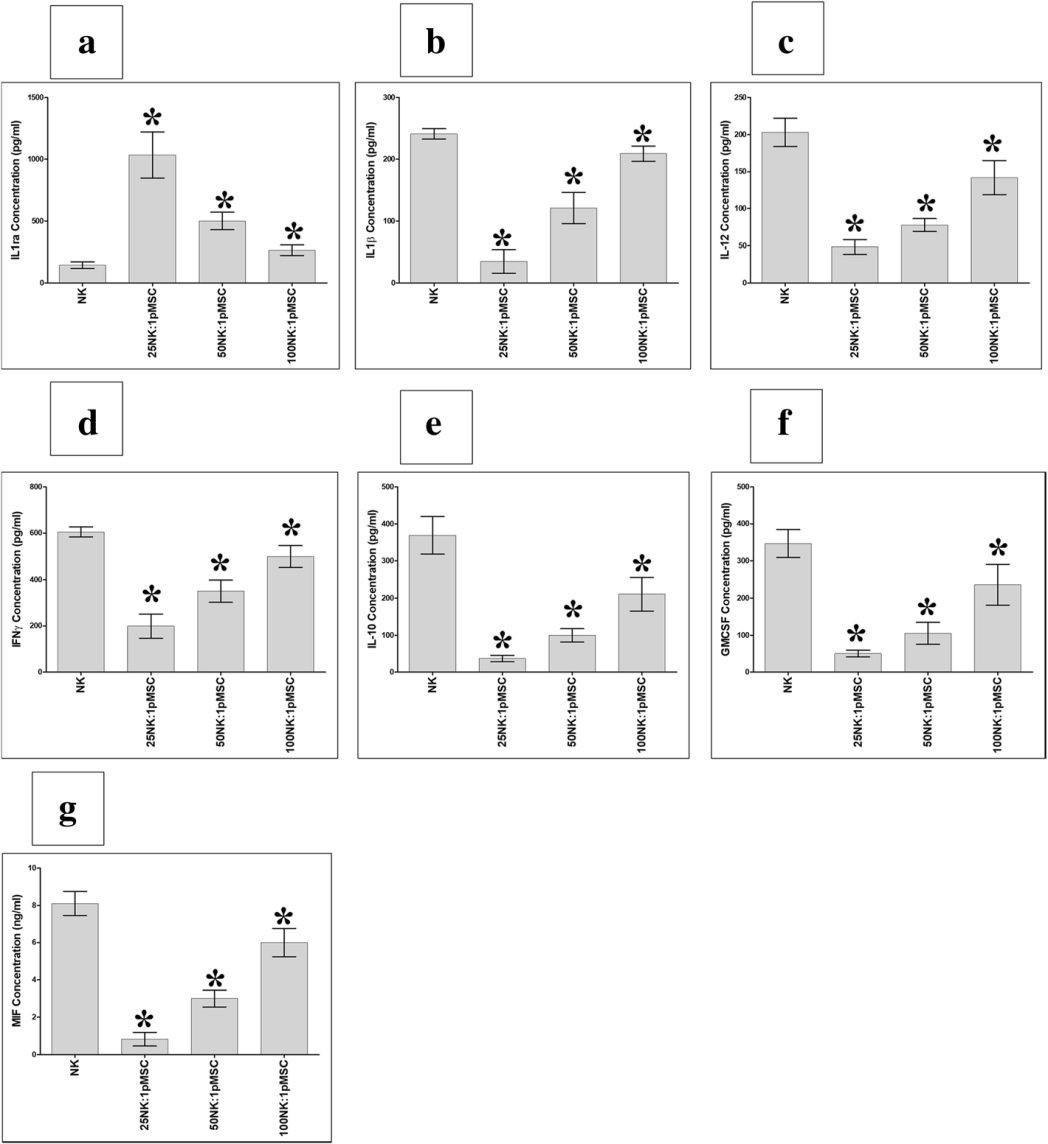
NK cell secretion of cytokines in the NK/pMSC cytolytic experiment where IL2-stimulated NK cells lysed pMSCs at 25:1, 50:1, and 100:1 NK to pMSC ratios. NK cell secretion of IL1ra, IL1β, IL10, IL12, IFNγ, GM-CSF, and MIF was evaluated using sandwich ELISA. Cytokine secretion profiles showed that pMSCs in the cytolytic experiment significantly increased the secretion of IL1ra (**a**), *P < 0.05, and significantly decreased the secretions of IL1β (**b**), IL-12 (**c**), IFNγ (**d**), GM-CSF (**e**), MIF (**f**), and IL10 (**g**), *P < 0.05, by NK cells after 24 h culture of IL-2-activated NK cell co-cultured with pMSCs as compared to IL-2-stimulated NK cells cultured alone (no pMSCs). Each experiment was carried out in duplicate using culture medium obtained from ten independent NK/pMSC cytolytic experiments where in each experiment NK cells and pMSC prepared from the peripheral blood of ten different healthy subjects and ten different normal human term placentae, respectively. Bars represent standard errors

The secretion of the cytokines IL1ra, IL1β, IL12, IFNγ, GM-CSF, MIF, and IL10 by NK cells is of critical importance for their function [29]. Therefore, we investigated whether pMSCs co-cultured with NK cells in the NK cells/pMSC cytolytic experiments, as described above, would alter the secretion of these cytokines by NK cells. Supernatants from the NK cells used in the cytolytic experiment were screened for the production of the seven cytokines listed above by ELISA. As shown in Fig. 8, the co-culturing of NK cells with pMSCs significantly increased the secretion of IL1ra and decreased the secretion of IFNγ, IL12, GM-CSF, IL1β, IL10, and MIF at all NK to pMSC ratios examined (*P* < 0.05). The modulatory effect of pMSCs on the secretion of these cytokines by NK cells was dose-dependent.

## Discussion

In this study, we investigated whether pMSCs interact with NK cells, thereby affecting the NK cell cytolytic activity and immune marker expression. Our results provide evidence that pMSCs are susceptible to lysis by NK cells and that this response is mediated by the interactions between NK activating receptors and pMSC ligands. pMSCs were also able to reduce the proliferation of IL-2-activated NK cells and of non-activated NK cells that were induced to proliferate by IL-2 (Fig. 1). These data are in agreement with previous studies that have demonstrated a reduction in NK cell proliferation after their interaction with bone marrow-derived MSCs (BMMSCs) [15, 30, 31]. However, our data contrast with our recent finding that MSCs from the maternal tissue of human placenta (DPMSCs) increase NK cell proliferation [19]. One obvious reason for this is that the original microenvironment (i.e., the niche) from which the MSCs were derived may regulate the functional activities of MSCs on NK cell proliferation. pMSCs are in direct contact with the fetal circulation and therefore exposed to low level of oxidative stress mediators during normal pregnancy [32, 33]. Similarly, MSCs in healthy bone marrow are exposed to low level of oxidative stress [34]. By contrast, DPMSCs in the maternal tissue of human placenta are exposed to increased level of oxidative stress during the course of human pregnancy [35, 36].

The mechanisms that underlie the pMSC-dependent reduction of NK cell proliferation are not known. However, this response is likely to be mediated by soluble factors secreted by pMSCs. This is because we have previously reported that pMSCs secrete molecules with anti-proliferative effects, such as prostaglandin E2 (PGE2) [25, 26]. The inhibition of PGE2 secretion by BMMSCs has been shown to reverse the anti-proliferative effect of BMMSCs on NK cells [31]. PGE2 might therefore also mediate the anti-proliferative effects of pMSCs on NK cells in a similar fashion. This possibility remains a question for future studies to address.

A previous study has also shown that NK cells are stimulated to lyse BMMSCs when BMMSCs express low levels of HLA class I molecules [15]. Our results show that although pMSCs expressed high levels HLA-E and HLA-ABC, they were nevertheless susceptible to being lysed by NK cells (Fig. 3). This finding therefore shows that the lysis of pMSCs by NK cells is not inhibited by HLA class I molecules as we recently reported that the cytolytic activity of NK cells against DPMSCs is independent of HLA class I molecules [19]. We further confirmed this by showing that the preconditioning of NK cells by pMSCs did not interfere with NK cytolytic activity against MCF-7 cells, which also express HLA-class I antigens (Fig. 5). These data are also in agreement with our previous study which demonstrated that DPMSCs do not interfere with NK cell lysis of MCF-7 cells [19]. However, our data contrast with a previous study, which reported that MCF-7 cell lysis by NK cells was decreased by preconditioning of NK cells with BMMSCs [30]. This difference in response could be attributable to the influence of the different placental MSCs and bone marrow MSCs niches in their source tissues, which might influence their effects on the cytolytic activity of NK cells.

pMSCs express low levels of NK cell-activating receptor ligands (Table 3). This was further confirmed by antibody-blocking experiments, which revealed that blocking the NK cell-activating receptors (NKG2D, DNAM-1, NKp30, NKp44, and NKp46) did not inhibit NK cells from lysing pMSCs (Fig. 4). By contrast, blocking the NK-activating receptor, CD69, substantially inhibited NK cell lysis of pMSCs (Fig. 4). This suggests that CD69 might play a role in mediating pMSC lysis by NK cells, although the co-culturing of NK cells with pMSCs reduced their expression of CD69 relative to control NK cells (Fig. 6a). The NK cell inhibitory receptor CD94/NKG2A was found to play no role in pMSC lysis by NK cells.

The preconditioning of NK cells by pMSCs also inhibited the expression of other activating receptors by NK cells, including NKG2D, NKp30, and NKp46 (Fig. 6). This suggests that the cytolytic activity of NK cells could be reduced by pMSCs. However, this appears not to be the case, since NK cells preconditioned by pMSCs were still able to lyse MCF-7 cells (Fig. 5). This suggests that other NK cell-activating receptors might be responsible for the lysis of MCF-7 cells by NK cells. In this regard, we found that the preconditioning of NK cells by pMSCs induced the NK cell expression of DNAM, which indicates that this activating receptor might mediate the lysis of MCF-7 cells by NK cells. We also found that NK cell expression of NKp30 was enhanced by preconditioning the NK cells with pMSCs, but only at a low ratio of NK cells to pMSCs. This suggests that NKp30 might also mediate NK cell cytolytic activity but only under certain conditions.

The lysis of pMSCs by NK cells questions the suitability of using pMSCs in regenerative medicine where pMSCs must function to repair injured or damaged tissue. However, pMSCs may contribute to tissue repair via paracrine mechanisms as we recently reported for DPMSCs [19]. pMSCs secrete numerous molecules that have reparative properties, such as IL-10, vascular endothelial growth factor (VEGF), transforming growth factor-β1 (TGFβ-1), B7-H4, and PGE2 [25, 26]. These molecules act on lymphocytes, including T, B, and NK cells, and other cell types to modulate their functions [22, 25, 26]. Consequently, these molecules are likely to mediate the functions of pMSCs in vivo. Future studies are therefore needed to elucidate the in vivo mechanisms that underlie the effects of pMSC functions on NK cells and on other immune or non-immune cells.

pMSCs were not lysed by resting, non-activated NK cells (Fig. 2b–e). Instead, their proliferation was decreased by NK cells (Fig. 2g, h). In contrast, when NK cells were activated with IL-2, this treatment enhanced their ability to lyse pMSCs (Fig. 3). These data demonstrate that inflammation, or an inflammatory milieu, increases the cytolytic activity of NK cells against pMSCs. This NK cell inflammatory phenotype is also increased by the exposure of these NK cells to pMSCs. We found that following co-culture with pMSCs, NK cells increased their expression of IL-18Rβ, while IFNγ R2 expression was reduced (Fig. 7a, e). It was noticeable that the ratio of pMSCs to NK cells affected the expression of some inflammatory molecules. A low pMSC to NK ratio increased the expression of IL-12 Rβ1 and IFNγ R1 by NK cells (Fig. 7b, c), while at high pMSC to NK ratios, IL-12Rβ1 expression was reduced (Fig. 7b). Our results indicate that, depending on their relative numbers, pMSCs can induce an inflammatory or anti-inflammatory phenotype in NK cells, although the mechanism underpinning this complex response is not known. The ratio of pMSCs to NK cells also affected the secretion by NK cells of both inflammatory and anti-inflammatory mediators (IFNγ, IL12, GM-CSF, IL1β, IL10, and MIF). However, pMSCs increased the secretion of IL1ra by NK cells, which is a naturally occurring anti-inflammatory cytokine that blocks the activity of IL-1 and has anti-tumor effects [37]. Our results suggest that pMSCs might enhance the anti-tumor activities of NK cells through IL1ra or via other mediators including IL12, IL-18, and IFNγ receptors and their corresponding cytokines (IL-18, IL-12, and IFNγ), which are known to be involved in the anti-tumor activity of NK cells [38–40]. This possibility is further supported by the finding that pMSCs induce NK cell expression of TLR3, at least at a high pMSC ratio. TLR3 has a role in mediating the anti-tumor activities of NK cells [41]. Therefore, our data suggest that pMSCs could be used to treat cancers by enhancing the anti-cancer activity of NK cells in vitro through different mechanisms that would involve IL-12, IL-18, and IFN-ɣ receptors, as well as TLR3 and IL1ra as we previously suggested for DPMSCs [19]. Before realizing this possibility, further studies are needed to elucidate the molecular mechanisms that underlie the enhancement of the anti-tumor activities of NK cells by pMSCs.

## Conclusion

Our data suggest that preconditioning NK cells with pMSCs stimulates NK cells to express inflammatory as well as anti-tumor molecules, which could be advantageous for cancer therapy.
